# Dramatic and sustained increase in HIV-testing rates among antenatal attendees in Eastern Uganda after a policy change from voluntary counselling and testing to routine counselling and testing for HIV: a retrospective analysis of hospital records, 2002-2009

**DOI:** 10.1186/1472-6963-10-290

**Published:** 2010-10-14

**Authors:** Robert Byamugisha, Thorkild Tylleskär, Mike N Kagawa, Saul Onyango, Charles AS Karamagi, James K Tumwine

**Affiliations:** 1 Referral Hospital, Department of Obstetrics and Gynaecology, Mbale Regional Referral Hospital, Mbale, Uganda; 2 Centre for International Health, University of Bergen, Bergen, Norway; 3 School of Medicine, Makerere University College of Health Sciences, Kampala, Uganda; 4 Uganda AIDS Commission, Kampala, Uganda

## Abstract

**Background:**

The burden of mother-to-child transmission of HIV in Uganda is high. The aim of this paper is to describe the experience of the first 7 years of the prevention of mother- to- child transmission of HIV (PMTCT) programme in Mbale Regional Hospital, Eastern Uganda, with particular reference to the lessons learnt in changing from voluntary counselling and testing (VCT) to routine counselling and testing (RCT) for HIV testing in antenatal services.

**Methods:**

The study was a retrospective analysis of the PMTCT records of Mbale Regional Referral Hospital, Uganda, from May 2002 to April 2009. The data on HIV testing of pregnant women and their male partners was extracted from the reports and registers using a standardized data extraction form, and data was analysed using descriptive statistics. Permission to conduct the study was obtained from School of Medicine, Makerere University College of Health Sciences; Uganda National Council of Science and Technology, and Mbale Hospital.

**Results:**

A total of 54 429 new antenatal (ANC) attendees and 469 male-partners accessed antenatal services at Mbale Regional Referral Hospital. There was a sustained, significant increase in HIV testing among new ANC attendees from 22% during the VCT period to 88% during the RCT period (*p *= 0.002), while among male partners, HIV testing increased from 88% to 100% (*p *= 0.010) However, the overall number of male partners who tested for HIV remained very low despite the change from VCT to RCT approach in HIV testing.

**Conclusions:**

Routine offer of antenatal HIV testing dramatically increased HIV testing in pregnant women and their partners in Uganda. Our findings call for further strengthening of the policy for routine HIV testing in antenatal clinics. Our study also showed that male partner HIV testing in antenatal clinics is low and this area needs further work through research and innovative interventions in order to improve male partner involvement.

## Background

Over the last decade, large efforts have been used to prevent mother-to-child transmission (MTCT) of HIV in Sub-Saharan Africa. The prevention of mother-to-child transmission of HIV (PMTCT) programme has evolved over the past decade and currently comprises of HIV counselling, HIV-testing, infant feeding counselling, and antiretroviral prophylaxis.

In Uganda, the burden of mother-to-child transmission of HIV in Uganda is high because of the large number of deliveries (1.3 million deliveries per year), almost universal breastfeeding [1] and the high [6.0%] antenatal HIV prevalence [2,3]. This implies that about 78 000 of the women who deliver annually are living with HIV. Hence without any interventions for PMTCT, about 30% of these women would pass the virus to their babies. This translates to about 23 400 infected children every year, at least half of would be prevented if there were a nation-wide PMTCT programme. The national PMTCT programme was launched as a pilot intervention in 8 hospitals in the year 2000, and was integrated into existing antenatal care services. The Government of Uganda in collaboration with UNAIDS, UNICEF and other partners scaled up the PMTCT programme to all districts by December 2004. The programme was introduced as a client-initiated approach where antenatal care clients were encouraged to undergo counselling and testing for HIV if they so wished. This approach is also known as 'opt-in' or voluntary counselling and testing (VCT).

The prevention of mother-to-child transmission of HIV package consisted of interventions designed to reduce the risk of HIV transmission during pregnancy, labour, delivery and during the postnatal period. The comprehensive PMTCT package comprises of the following:

• VCT that is integrated within the antenatal clinic services

• Quality antenatal, intra-natal and postnatal care services as well as follow-up support for both the mother and baby

• Antiretroviral (ARV) drugs for HIV-positive mothers and their babies

• Counselling and support on optimal infant and young child feeding

• Promotion of family and community support including the involvement of male partners and spouses

The ARV prophylaxis regimen was a single dose of *Nevirapine (NVP) *200 milligrams (mg) to the mother at onset of labour, and a single of *NVP *(2 mg/kilogram [kg] body weight), to the baby within 72 hours of birth. The infant feeding options to HIV infected mothers were either breastfeeding only for 3 months with abrupt weaning or replacement feeding if it was affordable, feasible, acceptable sustainable and safe (AFASS). By December 2005, the PMTCT programme was providing HIV counselling to about 35% and HIV testing to about 20% of all pregnant women in the country. Only 15% of the pregnant women living with HIV in the country were identified and about 10% of them accessed prophylactic antiretroviral drugs for PMTCT [4]

Following CDC and UNAIDS/WHO recommendations [5,6], there was a change in strategy in the year 2006. The provider-initiated approach that routinely tests all antenatal attendees for HIV was introduced in the PMTCT programme in Uganda in 2006. This approach is also known as 'opt-out' or routine counselling and testing (RCT). Concurrently, new guidelines on the use of ARV prophylaxis for PMTCT were issued. The ARV regimen included: *Zidovudine (AZT, ZDV) *300 mg given to the mother orally plus *Lamivudine (3TC) *150 mg twice a day starting from 32 weeks through out labour and boosted by a single dose of *NVP *200 mg at onset of labour. Then she continues with *AZT plus 3TC *twice a day for one week after delivery. The baby receives a single dose of *NVP *syrup (2 mg/kg body weight) within 72 hours of birth and *AZT *syrup (4 mg/kg body weight, twice a day for seven days. The HIV-infected mothers were recommended to either exclusively breastfeed for 6 months instead of 3 months or give replacement feeds if affordable, feasible, acceptable sustainable and safe.

In Uganda, about 94% of the women attend the antenatal clinic at least once during pregnancy but less than half (47%) of them receive 4 or more visits for antenatal care. Only about 17% of the women make their first antenatal visit in the first three months of pregnancy. However, a high proportion (41%) of women makes their first antenatal care (ANC) visit in the fourth or fifth months of pregnancy. The median gestational age at the first visit is 5.5 months, when the opportunity may have passed to diagnose problems early, provide treatment, and prevent further complications. About 41% of women deliver in a health facility and about 58% deliver at home [7]. The aim of this paper is to describe the experience of the past 7 years of the PMTCT programme in Mbale Regional Hospital, Eastern Uganda, with particular reference to the lessons learnt in changing from the VCT to the RCT strategy for HIV testing and counselling in antenatal services.

## Methods

The study was conducted at Mbale Regional Referral Hospital in Mbale district, Eastern Uganda. It was a retrospective analysis of data from hospital records on HIV counselling and testing of antenatal attendees in the hospital covering a seven year period (May 2002 to April 2009). The hospital is located in Mbale town approximately 240 kilometres North-East of Kampala city by road. The district had a population of over 720 000 in the year 2002, with an annual population growth rate of 2.5%. Like the rest of Uganda, 92% of the people live in the rural areas and are predominantly *Bagisu or Bamasaba*. The main language is *Lumasaba *and the main economic activity is subsistence farming. The literacy rate was 64% for men and 49% for women [8]. In 2003, the HIV prevalence was reported to be 5.6% [9]. The PMTCT services are offered at Mbale Regional Referral Hospital, Bufumbo Health Centre IV and some of health centre III units in the district. It is a regional referral hospital for 11 districts in Eastern Uganda and serves an estimated population of 1.9 million people. The hospital has a bed capacity of 380 and serves approximately 6 000-9 000 new antenatal attendees per year. The antenatal care services are provided daily except weekends. The average attendance is 50-60 pregnant women per day, including those who come for ANC return visits. The HIV testing policy is communicated to women attending ANC by either nurses/midwives or lay counsellors during the health talk given in the morning after the mothers' registration exercise in the clinic. All the health workers involved in the PMTCT programme underwent a basic two-week course on counselling for PMTCT and a one-week course on infant and young child feeding counselling, though some of them are now fully-trained professional counsellors. They also received a one-week refresher course on updates about HIV counselling and testing before the introduction of RCT in June 2006. Initially the counsellors offered one-to-one pre and post-test counselling, since the number of clients accessing the PMTCT services was low. However, as the number of antenatal attendees undergoing VCT increased, pre-testing counselling was given to small groups of about 5 clients (from January 2003) but individual post-test counselling was maintained. The pregnant women were encouraged to come for HIV testing with their partners but very few men showed up for VCT. However since introduction of RCT in June 2006, the service-providers give pre-test group counselling to large groups of ANC attendees, but offer one-to-one post-test counselling to the women. Couples who come for RCT are seen first before attending to the mothers who come alone as one way of encouraging couple attendance.

HIV testing is done on-site by the health staff in the antenatal clinic who have received a five-day training on HIV tests using rapid HIV testing kits. All the other routine antenatal tests are performed by the laboratory staff in the hospital's main laboratory nearby. A sequential HIV testing algorithm, with same day results, including three rapid tests is used on one blood sample: *Determine HIV 1/2 assay (Abbott Laboratories, Abbott Park, IL, USA) *for first screening; *STAT-PAK HIV 1/2 DIPSTICK assay (Chembio Diagnostic Systems Inc.) *as a second test and *Uni-Gold (Trinity Biotech, Wicklow, Ireland) *as a "tie-breaker". The ANC attendees are classified as uninfected if *Determine *is negative and as HIV-infected if both *Determine *and *STAT-PAK *tests are positive. Discordant *Determine *and *STAT-PAK *blood samples are tested using the *Uni-Gold *test. The HIV test result is reported as positive if the *Uni-Gold *test is positive or as negative if both *STAT-PAK *and *Uni-Gold *tests are negative. Since 2006 ANC attendees who test HIV-positive undergo CD4 cell count before being given appropriate treatment according to the national PMTCT guidelines [10].

Several registers are used to monitor and evaluate the performance of the PMTCT programme. The *VCT and HIV Register *is used during counselling and testing for HIV to collect data from all women who are counselled. The *Enrolment Register *is used to collect information from HIV positive mothers at the time of getting the antiretroviral drugs (accessed free of charge in the antenatal clinic or the labour ward). The *Delivery and Birth Register *is used to collect information from all HIV-positive women who have been enrolled on the programme, at the time when they are in labour and immediately after they have delivered. The *Follow-up Register *is used to collect data during the postnatal period and beyond while the mother and baby are being followed up till the bay is at least eighteen months of age. In addition, there are two summary registers, namely the *Antenatal Summary Register *and the *Delivery Summary Register*, for monthly antenatal activities as well as activities related to labour and delivery. Every month, the PMTCT coordinator in the hospital compiles a report about the PMTCT activities in the hospital, a copy of which is sent to the National PMTCT programme manager in the Ministry of Health headquarters.

We analysed the monthly PMTCT programme reports, the *VCT and HIV Registers, the Enrolment Registers and the Antenatal Summary Registers *in the antenatal clinic, and the *Delivery Summary Registers and HIV registers *in the maternity ward at Mbale Regional Referral Hospital covering a seven-year period (May 2002-April 2009). The data was extracted using a standardized data extraction form, and entered into an excel sheet. The variables of interest were: total number of new antenatal attendees per year, number of ANC attendees counselled about HIV, number of ANC attendees who underwent HIV-testing and number of ANC attendees who obtained a positive HIV-test result. The other variables were: number of male partners counselled for HIV, number tested for HIV and number of those who obtained a positive HIV-test result, number HIV-infected mothers and their infants who received ARV prophylaxis for PMTCT. The total number of new attendees and male partners who accessed the ANC and PMTCT services, and the total number of HIV-positive women and their infants that accessed ARV drugs (ARVs) was computed annually. The percentages of the ANC attendees or their male partners who were counselled and tested for HIV and of those who tested HIV-positive were calculated. The few pregnant women who knew that they were HIV positive at their first antenatal visit were recorded in the *VCT and HIV Registers *as having been counselled about HIV and infant feeding options, and we included them in the analysis among the total number HIV-positive ANC attendees. The *Student's t-test *was used to compare some selected indicators for PMTCT in the hospital before and after the policy change in antenatal HIV-testing approach. A *p-*value of < 0.05 was considered statistically significant.

Ethical clearance to conduct the study was obtained from the Research and Ethics Committee of the School of Medicine, Makerere University College of Health Sciences; the Uganda National Council of Science and Technology; and the National PMTCT programme, Ministry of Health, Uganda. Permission to use the hospital records was obtained from the Mbale Regional Referral Hospital administration through the local institutional review board.

## Results

### Overall results of HIV testing

From May 2002 to April 2009, a total of 54 429 new ANC attendees and 469 male-partners accessed antenatal services at Mbale Regional Referral Hospital. Of all the new ANC attendees, 42 754 (78.6%) were counselled about HIV, 28 108 (51.6%) were tested for HIV and 1713 (6.1%) of those tested received an HIV-positive result. During the same period, a total of 469 male partners received HIV counselling while 459 (97.9%) of them were tested for HIV and 40 (8.7%) of those tested were HIV positive.

### Trends of HIV testing in pregnant women

**Figure 1:**
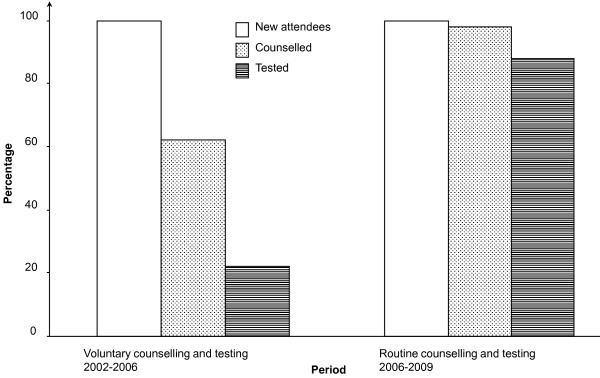
**Trends in HIV testing among new antenatal attendees, Mbale Regional Referral Hospital, 2002-2009**.

From May 2002 to May 2006 (VCT period), 6 570 (22.0%) out of 29 834 new ANC attendees were tested for HIV as compared to 21 538 (87.6%) out of 24 595 new ANC attendees who were tested during the RCT period from June 2006 to April 2009 (*p *= 0.002), with a corresponding increase in numbers of HIV-infected women identified antenatally (n = 1147, 5.3% sero-prevalence as compared with 566, 8.6% sero-prevalence in the VCT period, *p *= 0.012), Figure 1 and table 1.

**Table 1:** Selected indicators of prevention of mother-to-child HIV transmission at Mbale Regional Referral Hospital, 2002-2009

Indicator	Voluntary HIV testing period n (%)	Routine HIV testing period n (%)	*P*-value (two-tailed)
*New ANC attendees*			
Booked for ANC	29834	24595	-
Counselled for HIV	18583 (62.3)	24171 (98.3)	0.042*
Tested for HIV	6570 (22.0)	21538 (87.6)	0.002*
HIV positive	566 (8.6)	1147 (5.3)	0.012*
*Male Partners of the ANC attendees^a^*			
Counselled for HIV	80 (100)	389 (100)	0.012*
Tested for HIV	70 (87.5)	389 (100)	0.010*
HIV positive	15 (21.4)	25 (6.4)	0.112
*HIV- infected pregnant women*			
Used ARVs for PMTCT^b^	316 (55.8)	885 (77.2)	0.015*
Delivered in hospital	172 (30.4)	464 (40.5)	0.042*
Infants given ARVs for PMTCT^c^	184 (32.5)	451 (39.3)	0.050

* Statistically significant *p*-value of the independent-samples *t*-test at the 0.05 level

**^a ^**All male partners who accompanied their spouses for the antenatal care visit were counselled about HIV.

**^b ^**During the VCT period the HIV- infected women and their infants received single dose *Nevirapine (sdNVP)*, but in the RCT period the mothers received a combination regimen of *Zidovudine (ZDV, AZT) *and *Lamivudine (3TC) *from 32 weeks plus *sdNVP *at onset of labour. The infants received *sdNVP *syrup and *ZDV *syrup for one week. Women were started on HAART after 14 weeks gestation if they had a CD4 count of < 350 or WHO disease stage 3 or 4 clinically.

**^c ^**Some babies were brought to hospital for the ARVs within 72 hours of birth outside the hospital and some babies born in the hospital missed getting ARVs when out of stock.

**Figure 2:**
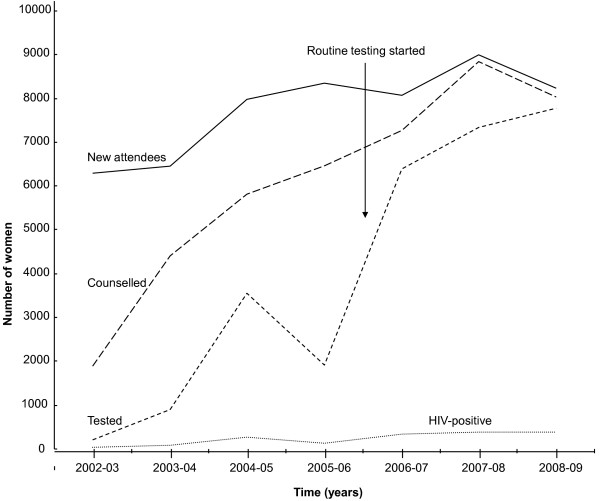
**Trend of HIV counselling, testing and sero-positivity among new antenatal attendees, Mbale Regional Referral Hospital, 2002-2009**.

During the period of May 2002 to April 2003, a total of 6 298 pregnant mothers attended their first antenatal visits. Of these, 1 903 (30%) were counselled and 209 (3%) were tested for HIV. A steady increase in pregnant mothers accepting voluntary counselling and testing (VCT) for HIV was observed during the first three years of the PMTCT programme, Figure 2 and table 2. From May 2004 to April 2005, out of 7 982 new ANC attendees, 3 546 (44%) accepted HIV testing. However in the following year (May 2005 - April 2006), 1 915 (22.9%) out of 8 358 pregnant mothers were tested for HIV though 5 811 (73%) new ANC attendees had been counselled.

**Table 2:** Antenatal HIV counselling and testing among new attendees at Mbale Regional Referral Hospital, 2002-2009

Period	HIV testing Approach	New ANC attendees N	Counselled n (%)	Tested n (%)	HIV-positive n (%)
May 02 - April 03	VCT	6 298	1 903 (30.2)	209 (3.3)	45 (21.5)
May 03 - April 04	VCT	6 468	4 407 (68.1)	900 (13.9)	101 (11.2)
May 04 - April 05	VCT	7 982	5 811 (72.8)	3 546 (44.4)	288 (8.1)
May 05 - April 06	VCT	8 358	6 462 (77.3)	1 915 (22.9)	132 (6.9)
					
May 06 - April 07	RCT	8 079	7 286 (90.2)	6 393 (**79.1)**	356 (5.6)
May 07 - April 08	RCT	8 999	8 851 (98.4)	7 353 (**81.7)**	387 (5.3)
May 08 - April 09	RCT	8 245	8 034 (97.4)	7 792 (**94.5)**	404 (5.2)

In the first year after the policy change from VCT to RCT, there was a dramatic increase in the number of mothers who were tested for HIV. Out of 7 286 new ANC attendees, 6 393 (79%) of them were tested for HIV. The number of mothers accepting an HIV-test rose to 95% (7 792 mothers out of 8 245) in the period from May 2008 to April 2009, table 2. The increase in coverage of antenatal HIV testing among the pregnant women led to a corresponding significant increase in the use of PMTCT interventions, table 1.

### Trends of HIV testing in men

During the VCT period, out of 80 male partners that accompanied their spouses to the antenatal clinic, 70 (87.5%) of them were tested for HIV compared with 389 male partners (100%) who escorted their spouses for antenatal care and accepted HIV testing (*p *= 0.010), with a corresponding increase in the numbers of HIV-infected male partners identified in the RCT period (n = 25, 6.4% sero-prevalence as compared with 15, 21.4% sero-prevalence in the VCT period, *p *= 0.103), table 1 and table 3. In comparison with the new ANC attendees, the number of male partners who underwent counselling and testing for HIV at the antenatal clinic in Mbale Regional Referral Hospital remained very low despite the change from VCT to RCT approach in HIV testing, table 1 and 3.

**Table 3:** Male partners counselled and tested for HIV at Mbale Regional Referral Hospital, 2002-2009

Period	HIV testing approach	Male partners^a ^counselled N	Male partners tested n (%)	Male partners HIV-positive n (%)
May 02 - April 03	VCT	8	7 (87.5)	3 (42.9)
May 03 - April 04	VCT	6	6 (100)	1 (16.7)
May 04 - April 05	VCT	32	28 (87.5)	8 (28.6)
May 05 - April 06	VCT	34	29 (85.3)	3 (10.3)
				
May 06 - April 07	RCT	103	103 (100)	11 (10.7)
May 07 - April 08	RCT	92	92 (100)	9 (9.8)
May 08 - April 09	RCT	194	194 (100)	5 (2.6)

^a ^All male partners who accompanied their spouses for antenatal care were counselled (100%) and most of them were tested for HIV in the antenatal clinic.

## Discussion

Our study showed that the policy change from VCT to RCT dramatically increased the number of mothers tested for HIV. This change was sustained over the 3 years of observation. These findings are similar to those reported elsewhere in Africa [11]. In rural Malawi, the number of pregnant women who accepted HIV-testing increased from 78.7% to 98.8% after changing from VCT to RCT testing approach for HIV[12]. A study from an urban area in Zimbabwe found that 99.9% of the ANC attendees underwent HIV-testing during the first 6 months of routine HIV-testing compared to 65% of the women during the last 6 months of opt-in testing[13]. A study conducted in Botswana demonstrated an increase in HIV testing rate from 75% in the last 3 months of VCT period to 91% in the first 4 months of RCT period [6].

The high proportion of routine HIV-testing in our study could be attributed to several factors. The new ANC attendees were less fearful of accepting HIV-testing because the opt-out approach was perceived by their partners and families as "standard of care" given to all pregnant women in the antenatal clinic. In addition, the availability of rapid HIV-testing in the clinic and the giving of same-day HIV test results may have contributed to the high proportion of HIV-testing. The refresher courses given to the ANC providers/counsellors with the launch of RCT in June 2006 may have equipped them with improved HIV counselling and testing skills, and thus may have had a positive impact on the testing during the RCT period. Our study has also shown that the high coverage of antenatal HIV-testing after policy change significantly identified more HIV-infected pregnant women who easily accessed the free antiretroviral drugs (ARVs) in the antenatal clinic and the maternity ward. A similar finding was reported in study in Zimbabwe [13].

During the first 3 years of the VCT period (May 2002 - April 2005), there was a substantial increase in HIV testing among the antenatal attendees from 3.3% to 44%, but testing coverage halved in the following year (May 2005/April 2006). This decline was largely due to inadequate supplies of HIV-test kits and sundries during that period and the HIV-test tests were completely out of stock in the months of April and May 2006, Figure 2. During those two months about 1400 new attendees visited the antenatal clinic but could not be offered HIV-testing services, leading to missed opportunities as has been documented elsewhere [14-16].

The apparent higher prevalence of HIV in the VCT period is likely to be attributed to selection bias. It is probable that the ANC attendees who perceived themselves to be at higher risk of HIV infection were the ones that most likely opted to test for HIV voluntarily. Coupled with the small number of pregnant women that were tested for HIV, this may partly explain the higher prevalence of HIV during the VCT period. The shift from one-to-one pre-test counselling to group pre-test counselling seems not to have impacted on the testing rates since there was a substantial increase in antenatal HIV-testing rates from 14% to 44% in the periods May 2003/April 2004 and May 2004/April 2005 respectively.

Our study showed that the number of male partners who tested for HIV at the antenatal clinic was low (below 5%) compared to the total number of the ANC attendees despite the change from "opt-in" to "opt-out" approach in HIV-testing. Poor male partner involvement in PMTCT programme has been documented elsewhere in Africa, and studies in Tanzania, Kenya and Zambia [17,7] have reported HIV testing rates among male partners of 9% to 16%. Male partner involvement is essential for improving PMTCT outcomes [17-19]. Thus the low rate of HIV-testing among the male partners remains a major challenge to the PMTCT programme in Uganda. Overall, 98% of the male partners of the new ANC attendees were tested for HIV. During the "opt-in" period, 88% of the male partners accepted HIV testing while 100% accepted HIV testing in the "opt-out" period. Although the number of male partners in our study was small, the opt-out approach appears to increase male partner testing for HIV.

The study revealed that the average prevalence of HIV among the new ANC attendees over the 7-year study period was 6.1%. Similarly, HIV prevalence rates of 6.0% and 5.9% have been reported by Musinguzi et al and the Uganda Demographic and Health Survey respectively [2,3]

Our study had a number of limitations. It was a retrospective analysis of routinely collected data in the antenatal clinic and made comparisons between two different time periods. Accordingly, the study may have suffered from selection bias arising from differential selection of the participants, and from confounding due to inability to measure and control for potential confounders concurrently. For example, it was not possible to measure and control for other possible factors like media influence that may have contributed to the increase in HIV testing that was observed in the study. The change in HIV testing from VCT to RCT coincided with a period of wide availability of free ARVs especially for treatment. This may have been a potential confounder since the ANC attendees knew they could easily access the required ARVs in case they were found to be HIV positive. It is also possible that the policy change in HIV testing strategy may have introduced some measurement bias but this is likely to have been minimal. In addition, there was missing data and limited demographic data in the records that may also have impacted on the study findings. Finally, the small number of male partners who accessed the PMTCT services may also have affected the validity of the data on male partners in the study.

## Conclusions

Despite these limitations, our study documented a dramatic and sustained increase in antenatal HIV testing among pregnant women and their partners in Mbale, Eastern Uganda following the change from VCT to RCT. Our findings call for further strengthening of the policy for routine HIV testing in antenatal clinics. Our study also showed that male partner HIV testing in antenatal clinics is low and this area needs further work through research and innovative interventions in order to improve male partner involvement.

## Competing interests

The authors declare that they have no competing interests.

## Authors' contributions

RB participated in the conception, design, and implementation of the study, statistical analysis, interpretation and drafting of the manuscript. CASK participated in statistical analysis and interpretation, and the drafting on the manuscript. TT participated in the conception and design of the study, interpretation and drafting the manuscript. MK participated in study conception, design and drafting of the manuscript. SO participated in interpretation and drafting of the manuscript. JKT participated in the study conception, design, statistical analysis, interpretation and drafting of the manuscript. All authors read and approved the final manuscript.

## Pre-publication history

The pre-publication history for this paper can be accessed here:

http://www.biomedcentral.com/1472-6963/10/290/prepub
